# Innovative antimicrobial coating of titanium implants with iodine

**DOI:** 10.1007/s00776-012-0247-3

**Published:** 2012-07-18

**Authors:** Hiroyuki Tsuchiya, Toshiharu Shirai, Hideji Nishida, Hideki Murakami, Tamon Kabata, Norio Yamamoto, Koji Watanabe, Junsuke Nakase

**Affiliations:** Department of Orthopaedic Surgery, Graduate School of Medical Science, Kanazawa University, 13-1 Takaramachi, Kanazawa, 920-8641 Japan

## Abstract

**Background:**

Postoperative infection associated with implants remains a serious complication in orthopedic surgery. Several biomaterial surface treatments have been proposed as a means of reducing the incidence of implant-associated infections. In this study, a clinical trial was performed using an iodine-supported titanium that suppresses the microbial activities.

**Methods:**

A total of 222 patients with postoperative infection or compromised status were treated using iodine-supported titanium implants. The mean age of the patients was 49.4 years (range 5–85 years). One hundred twenty-seven patients were male and 95 were female. In 158 patients, iodine-supported implants were used to prevent infection, such as compromised hosts and conditions, and in 64 patients to treat active infection. White blood cell (WBCs) and C-reactive protein (CRP) levels were measured pre- and postoperatively in all patients. To confirm whether iodine from the implant affected physiological functions, thyroid hormone levels in the blood were examined. Both examinations were conducted sequentially for a year. Radiological evaluations were performed regularly after the operation. The chronological changes of the iodine amount were evaluated using half pins removed after completion of external fixation.

**Results:**

The mean follow-up period was 18.4 months (range 3–44 months). Acute infection developed in three tumor cases among the 158 patients on preventive therapy. All three recovered without removal of the implants. Infection was cured in all 64 patients with infection. Median WBC levels were in the normal range, and median CRP levels returned to <0.5 within 4 weeks after surgery. Abnormalities of thyroid gland function were not detected. None of the patients experienced loosening of the implants. There were two patients with mechanical implant failure, which was treated by re-implantation. Excellent bone ingrowth and ongrowth were found around all hip and tumor prostheses. One year later, the amount of iodine on external fixation pins remained about 20–30 %.

**Conclusions:**

Iodine-supported titanium implants can be very effective for preventing and treating infections after orthopedic surgery. Cytotoxicity and adverse effects were not detected.

## Introduction

Surgical site infection associated with implants remains a serious and common complication in orthopedic surgery. The infection rate is 2.2 % after primary total hip arthroplasty [1] and 2.0 % following spinal operations [2], despite strict antiseptic operative procedures, which include systemic prophylaxis. Infection rates of 13 % have been described for endoprosthetic replacement of large bone defects after tumor resection, while external fixation leads to infection in up to 70 % of cases [3, 4]. Various biomaterial surface treatments have been developed to improve prophylaxis against implant-related infections. There have been investigations of the covalent attachment of polycationic groups [5], implantation of Ca+, N+ and F+ ions [6], impregnation and loading of chitosan nanoparticles with antimicrobial agents [7]. Other treatments of implant surfaces are drug-loaded polymers, quaternary ammonium compounds, human serum albumin and silver ions [8–11]. The antimicrobial properties of silver have led to its use as a coating for medical devices [12, 13]. While various coatings have been devised using silver salts or ion beam implantation of metallic silver, they have produced either disappointing clinical results [14–16] or further complications [17, 18]. The shortcomings of these proposed techniques are limited chemical stability, local inflammatory reactions due to material composition and a lack of controlled release kinetics from the coatings.

In this study, we used titanium implants with surfaces that were modified using anodization. Biocompatible metal-oxide implants have current clinical applications in orthopedic and dental implants [19]. As a biocompatible material, titanium and its alloys, particularly Ti–6Al–4V, are extensively used in orthopedic and dental implants. The excellent biocompatibility of titanium is reportedly attributable to the stable oxide that readily forms on its metal surfaces [20]. The composition of highly adhesive anodic oxide films that form through anodization is dependent on the electrolyte composition [21]. Povidone-iodine can also be used as an electrolyte, resulting in the formation of an adhesive, porous anodic oxide with the antiseptic properties of iodine [22]. Iodine is a component of thyroid hormones and is the heaviest essential element needed by all living organisms. Our previous studies indicate that iodine-supported titanium has antibacterial activity, biocompatibility and no cytotoxicity [22].

## Patients and methods

This study was approved by the institutional review board at the University of Kanazawa. Between July 2008 and June 2011, 222 patients with postoperative infection or compromised status were recruited into a prospective cohort study and treated with iodine-supported implants. Informed consents were obtained from the patients and their family. They were informed that new antibacterial implants were used for prophylaxis and treatment of postoperative infections.

The mean age of the patients was 49.4 years (range 5–85 years). One hundred twenty-seven patients were male and 95 were female. Patients were tested for iodine allergies with patch tests before surgery. The diagnoses included 95 cases of tumor (42.8 %), 34 cases of limb deformity (15.3 %), 29 cases of degenerative disease (13.1 %), 27 cases of osteomyelitis (12.2 %), 24 cases of pseudoarthrosis (10.8 %) and 16 cases of fracture (7.2 %). Iodine-supported implants were used to prevent infection for 158 patients (prevention group) who were likely to be infected, such as those with cancer, diabetes mellitus, and under steroid administration or chemotherapy, as well as those with compromised conditions such as open fracture (Gustilo type 2, 3-a, 3-b, 3-c) and long-term external fixation; and to treat active infections (*Staphylococcus*, *Escherichia coli*, fungus, etc.) in 64 patients (treatment group). Thirty-eight patients underwent one-stage revisions, while 26 needed two-stage revisions. Postoperatively, all patients received antibiotics intravenously for 3–7 days, followed by oral administration until wound healing was achieved.

**Fig. 1:**
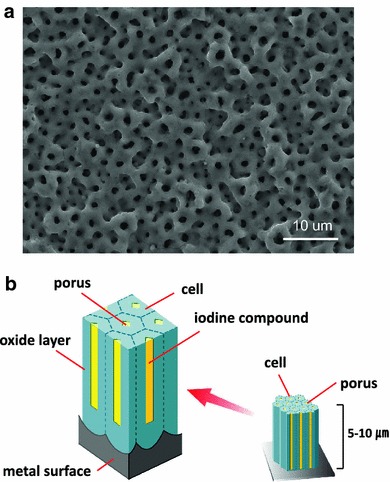
Electron micrograph of the oxide layer: more than 50,000 pores/mm^2^ (**a**). Diagram of the anodizing porous membrane (**b**)

The iodine supports were produced by the Chiba Institute of Technology (Narashino, Japan) using a technique described by Hashimoto [23]. The thickness of the anodic oxide film was between 5 and 10 μm, with more than 50,000 pores/mm^2^ with the capacity to support 10–12 μg/cm^2^ iodine (Fig. 1a, b). Postoperative radiological evaluations were performed regularly, during which time loosening and failure of the implants was checked by two specific radiological specialists (HT and TS). Moreover, the chronological changes of the iodine amount were evaluated using half pins removed after completion of external fixation.

White blood cells (WBCs) and C-reactive protein (CRP) were measured pre- and postoperatively in all patients. In patients with implant infections, a cure with no clinical signs of inflammation, a normal WBC level and a negative CRP were assessed by the treating clinician at the date of the last available follow-up. The levels of thyroid hormones in the blood, thyroid-stimulating hormone (TSH), free triiodothyronine (FT3) and free thyroxine (FT4) were examined to determine if iodine from the implant influenced physiological activities. Both examinations were conducted regularly for 1 year.

## Results

**Fig. 2:**
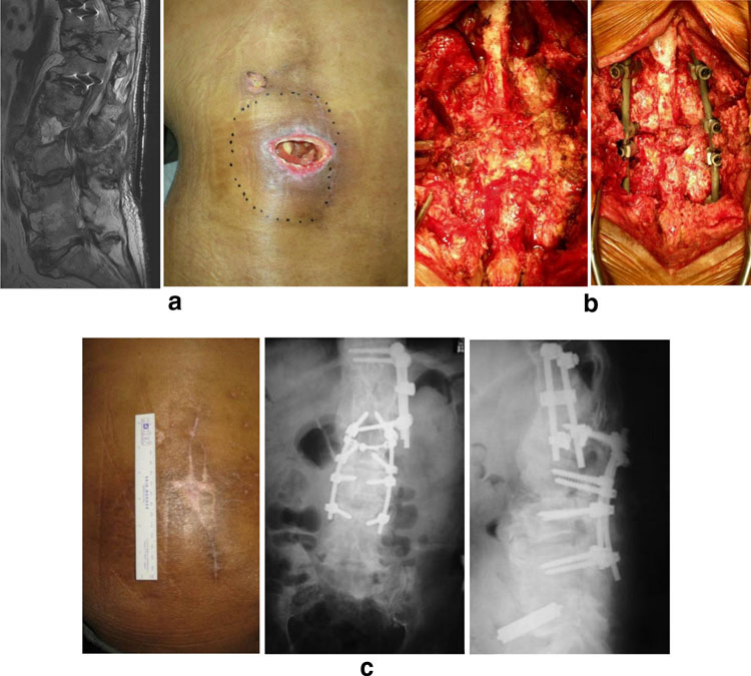
Case of a 57-year-old man with pyogenic spondylitis due to cord injury. Spinal pus sinus (**a**). Debridement was performed with primary instrumentation using iodine-supported titanium (**b**). The infection was cured and the fistula was closed after surgery (**c**)

**Fig. 3:**
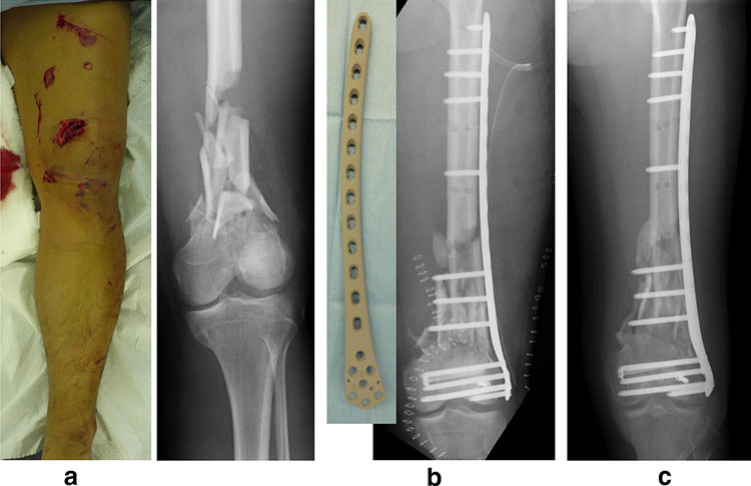
Case of a 42-year-old man. Gustilo grade IIIa open fracture (**a**). Primary internal fixation using iodine-supported titanium plate (**b**). No sign of infection and good callus formation at 4 months after surgery (**c**). Iodine did not inhibit bony union

**Fig. 4:**
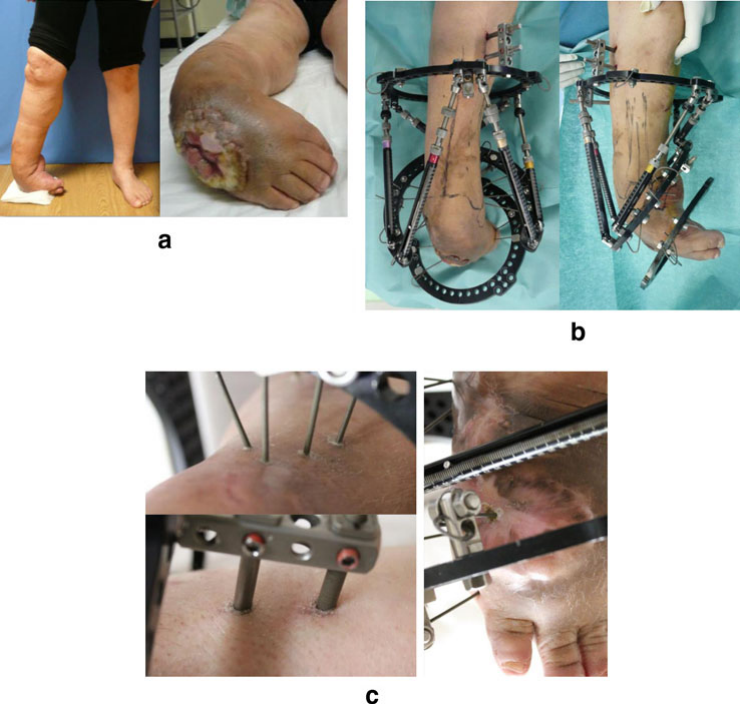
Case of a 64-year-old woman with a right clubfoot due to polio. Right clubfoot and ulceration with MRSA infection (**a**). External fixation performed to correct the right foot deformity (**b**). The iodine-supported pin tract site was still clean during the follow-up period (**c**)

**Fig. 5:**
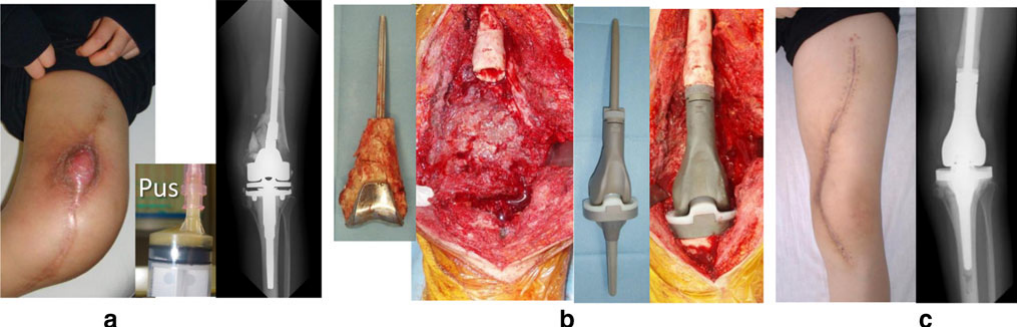
Case of a 19-year-old woman with osteosarcoma of the left femur. Postoperative coagulase-negative staphylococcus infection (**a**). Primary revision surgery using iodine-supported tumor prosthesis (**b**). No sign of infection after surgery (**c**). CRP was 0.1 mg/dl and WBC was 4,400/μl 10 months later

**Fig. 6:**
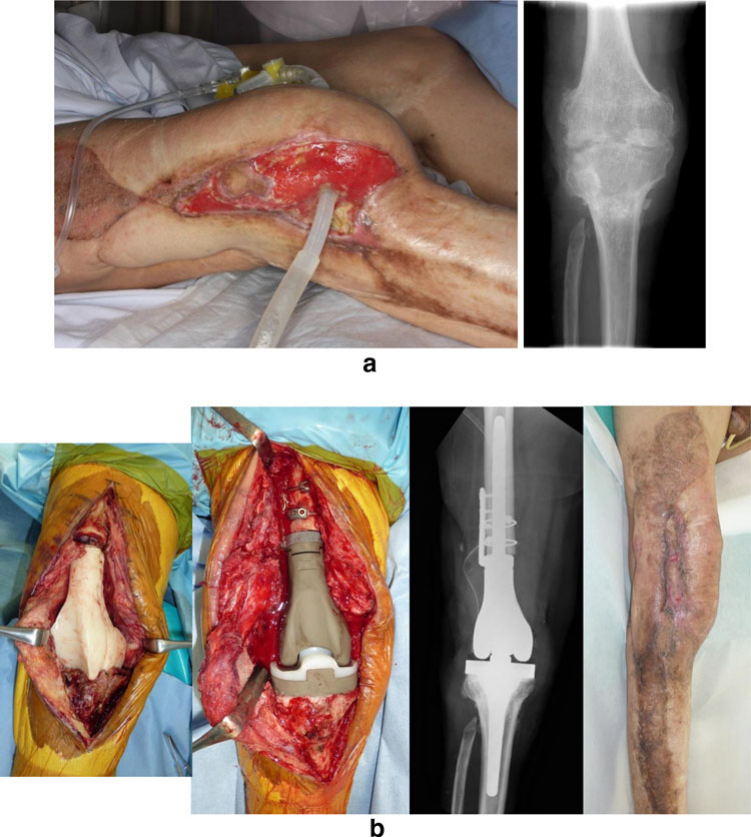
Case of a 74-year-old man with long-lasting MRSA arthritis of the right knee joint due to an open fracture in childhood. Continuous irrigation and suction (**a**). Secondary revision surgery using an iodine-supported tumor prosthesis; no sign of infection 15 months after surgery (**b**)

**Fig. 7:**
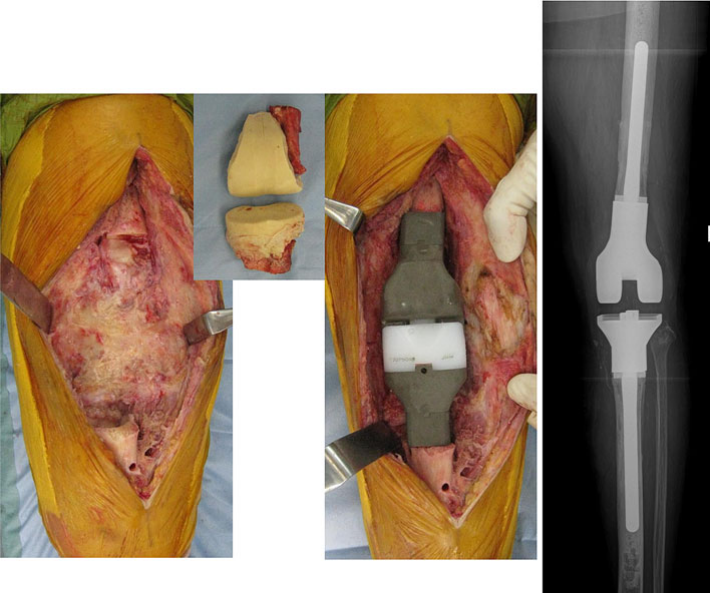
Case of a 72-year-old woman with refractory fungal arthritis after left total knee arthroplasty. Secondary revision surgery using iodine-supported prosthesis. The knee fungal arthritis healed following surgery (24 months)

The mean follow-up period was 18.4 months (range 3–44 months). The following types of implants were used: 82 spinal instrumentations (Fig. 2), 55 plates for osteosynthesis (Fig. 3), 36 external fixations (pins and wires) (Fig. 4), 32 tumor prostheses (Figs. 5, 6), 10 hip prostheses, 4 knee prostheses (Fig. 7), 2 nails and 1 cannulated screw. Acute infection developed in three tumor cases (1.9 %) among the 158 patients under preventive care. In one patient who had synovial sarcoma of the spine, surgical site infection occurred during bone-marrow suppression due to chemotherapy. The infection was cured by debridement and removing only the Marlex mesh while leaving the iodine-coated implants in place. One case with osteosarcoma of the proximal femur received a prosthetic reconstruction, while another received a total en bloc spondylectomy for a metastatic spinal tumor. The two infected cases were also cured by intravenous administration of antibiotics only, without removal of the implants.

The 64 treatment cases that underwent one-stage or two-stage revision surgery recovered without additional surgery. Among patients with active infection, one showed late hematogenous infection 2 years after revision surgery and one had a suspected iodine allergy. In all cases, there were no signs of infection at the time of the last follow-up.

**Fig. 8:**
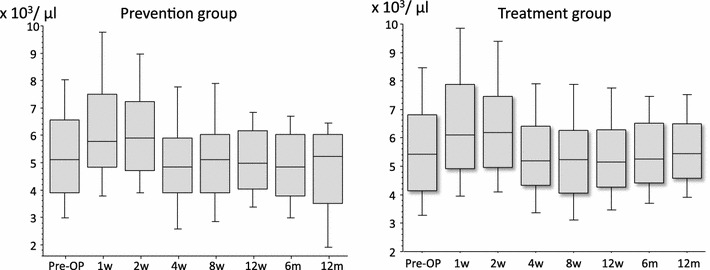
The levels of WBC/μl in the prevention group and the treatment group: In both groups, although WBC levels tended to be high 2 weeks after the operation, median WBC levels were in the normal range

**Fig. 9:**
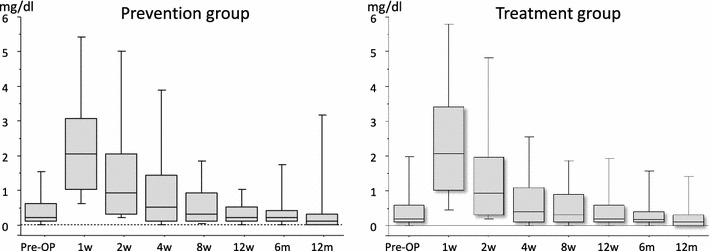
The CRP levels (mg/dl) in the prevention group and the treatment group: In both groups, median CRP levels returned to <0.5 within 4 weeks after surgery

**Fig. 10:**
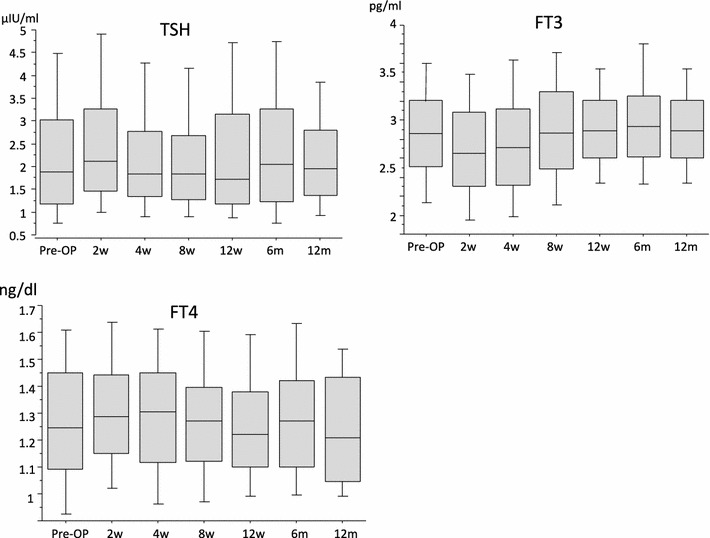
The TSH levels (μIU/ml), FT3 levels (pg/ml) and FT4 levels (ng/dl), which are thyroid hormone levels: TSH, FT3 and FT4 levels were in the normal range during the study period

In the treatment group, median WBC levels were in the normal range (Fig. 8), and median CRP levels returned to <0.5 within 4 weeks after surgery (Fig. 9). In the prevention group, these levels showed the same tendency (Figs. 8, 9). Abnormalities of thyroid gland function were not detected (Fig. 10).

**Fig. 11:**
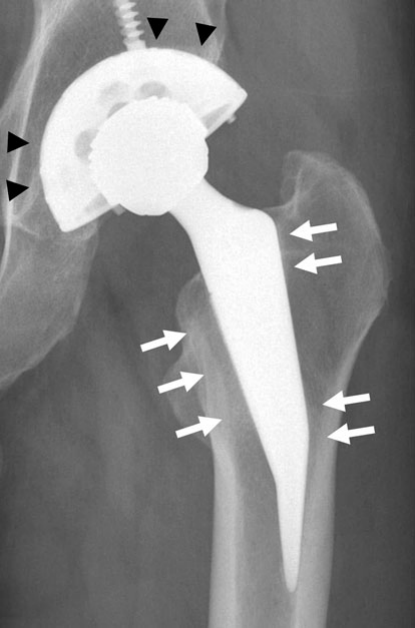
Excellent bone ingrowth (*filled triangle*) and obvious spot welds (*upward arrow*) were found around the hip prosthesis

None of the implants loosened during the follow-up period. However, two patients (0.9 %) experienced mechanical implant failure and were treated by re-implantation and bone grafts. Radiograms revealed excellent bone ingrowth and ongrowth around hip and tumor prostheses (Fig. 11). Redness or discoloration of the wounded area was not observed at the latest follow-up.

**Fig. 12:**
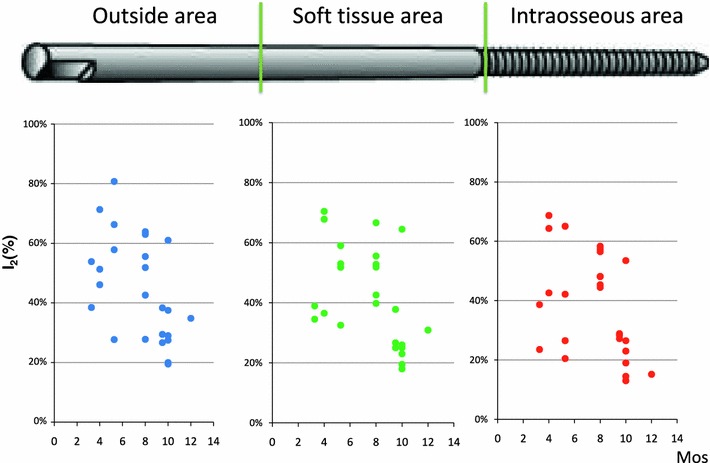
Iodine amount of half pin surface removed after treatment. Iodine was measured by X-ray fluorescent analysis (*n* = 25)

The amount of iodine on the removal external fixation pins decreased by about 50 % after half a year and 1 year later decreased to 20–30 % (Fig. 12).

## Discussion

The results of this clinical trial suggest that iodine-supported titanium implants can be very effective in the prevention and treatment of infections after orthopedic surgery. Implant surgeries are used in almost all fields of modern medicine and are associated with a definitive risk of bacterial infection. Staphylococci account for the majority of infections of both temporarily and permanently implanted orthopedic devices. Although surgical site infection rates in orthopaedic surgery are generally about 2 % [1, 2], it is more than 10 % in compromised hosts, and in patients with open fracture and external fixation [3, 4, 24, 28]. In this study, postoperative infection occurred in 3 of 158 (1.9 %) prophylaxis cases. All three were cancer patients. The 1.9 % infection rate was lower than the historical infection rate. Because drug resistance can prevent systemic administration of antibiotics from providing effective treatment for implant infections, it is important that the coating of the implant possesses local antibacterial activity. In order to reduce the incidence of implant-associated infections, several biomaterial surface treatments have been proposed [5–13]. There have also been reports on antibacterial alloy implants [25, 26]. In particular, silver has raised the interest of many investigators because of its good antimicrobial action and low toxicity [11–13], although some toxicity against human cells has been observed [14, 18]. It was reported at the “29th Annual Meeting of the European Bone and Joint Infection Society” in 2010 that 33 % (4/12) of patients classified as high-risk types, with chronic infections and major bone loss, developed localized cutaneous argyria after receiving silver-coated megaprostheses. In our opinion, silver-coated implants can present many problems clinically, such as cytotoxicity and no osteoconduction. Implants have also been coated with antiseptics such as chlorhexidine [27].

**Fig. 13:**
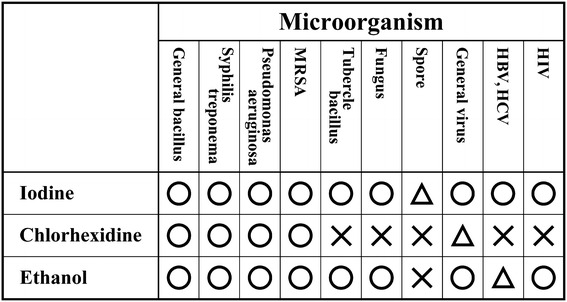
Antimicrobial spectrum of iodine

Iodine is also an antiseptic agent. However, there are no reports of the use of iodine-treated implants. This study is the first clinical trial of an iodine-coated implant. By an oxidizing action, iodine denatures protein and kills a microorganism. The antibacterial spectrum of iodine is very broad, acting not only on general bacteria but also viruses, tubercle bacilli and fungi (Fig. 13). From a basic experiment, it is enough to inhibit the growth of *Staphylococcus* and *Escherichia coli* completely if the surface of the titanium implants has an iodine concentration of 2–3 μg/cm^2^ (data not shown). In addition, iodine does not cause phenomena such as drug resistance induced by the administration of antibiotics. Moreover, iodine is a trace metal and an essential component of the thyroid hormone. If iodine is released from the implant, it is biologically safe because it can be excreted by the kidneys. In this study, TSH, FT3 and FT4 levels were in the normal range during the study period. Therefore, abnormalities of thyroid gland function were not detected.

It is a key point in this study that all three infected patients in prevention cases recovered without removing the implants even if infection occurred. It is usually difficult to cure infections without removing the implants if an implant-associated infection arises. However, we propose that recovery was possible in the presence of the iodine-coated implants because iodine-supported implants have antimicrobial effects. Therefore, these antibacterial iodine-coated implants are an innovative breakthrough.

Based on the medical literature, the successful eradication of a total joint replacement infection with a two-stage re-implantation protocol is over 90 %, while the success rate with a one-stage protocol is approximately 80 % [29]. In this study, we used iodine-supported implants in 56 patients for treating infection. However, reactivation of infection was not seen in either one-stage or two-stage revisions. The treatment strategy for postoperative infection was to perform two-stage revision surgery for active infections of extremities (patients with active discharge from sinuses or abscess formation), and one-stage revision surgery for inactive or already subsided infections. One-stage revision was performed as an exception for conducting chemotherapy to lung metastasis, although the osteosarcoma case (Fig. 5) had active infection. All patients with spinal infection were treated by one-stage revision surgery. The principles of revision are the removal of all possible infected tissue and bone with radical debridement. Antibiotics are systemically administered in conjunction with an antibiotic-loaded cement.

Median WBC levels remained below normal range (<8,000/μl) throughout the study, and CRP levels showed an increase within 4 weeks after surgery in both the treatment group and prevention group. The normalization of CRP was delayed by a postoperative reaction and the effects of an underlying disease such as cancer, diabetes and rheumatoid arthritis.

Bone conduction is reportedly not possible on certain materials such as silver and copper [30]. In this study, excellent bone ingrowth and ongrowth were shown around hip and tumor prostheses on radiograms. There were no signs of bone absorption or inhibition of bone formation. Previous studies have demonstrated that iodine-supported implants have excellent osteoconduction and good biocompatibility [22]. We think that the osteoconductive effects were reinforced according to the porous structure of the oxide layer.

Implant-associated infections were either prevented or cured in all patients by the iodine coating. Further studies with a longer follow-up period and with all replacement sites will follow. We plan to compare the prospectively collected data for the iodine-supported titanium group with retrospectively collected data for a titanium group to ascertain how long the iodine advantage is maintained in the body. At present, it is clear that 20–30 % of iodine remained on external fixation pins that were removed after 1 year (Fig. 12). We plan to measure the change in the amount of iodine for every kind of implant or insertion. The amount of iodine will be determined by the thickness of the oxide layer.

Regarding the limitation of this study, the infection rate of the prevention group was compared to a historical control. Therefore, a multi-center prospective randomized clinical trial on a large scale is necessary to demonstrate the statistical significance of the infection rate in the future.

## Conclusions

A clinical trial of iodine-supported titanium implants was performed with no detection of cytotoxic or adverse effects. The titanium was implanted at infectious lesions with no problems, as infections subsided because of the antibacterial activity of the implant. Moreover, iodine-supported implants worked to prevent postoperative infections in patients with compromised status. Therefore, the povidone-iodine coating is promising and can be applied to all titanium implants for preventing and treating infections following orthopedic surgery.
